# Influence of Harvest Time and Malaxation Conditions on the Concentration of Individual Phenols in Extra Virgin Olive Oil Related to Its Healthy Properties

**DOI:** 10.3390/molecules25102449

**Published:** 2020-05-24

**Authors:** Panagiotis Diamantakos, Triada Giannara, Maria Skarkou, Eleni Melliou, Prokopios Magiatis

**Affiliations:** Laboratory of Pharmacognosy and Natural Products Chemistry, Department of Pharmacy, National and Kapodistrian University of Athens, Panepistimiopolis Zografou, Athens 15771, Greece; totisdiam@hotmail.com (P.D.); tr.giannara@outlook.com.gr (T.G.); maria_skarkou@outlook.com.gr (M.S.); emelliou@pharm.uoa.gr (E.M.)

**Keywords:** quantitative nuclear magnetic resonance, olive oil, malaxation, harvest, phenols, oleocanthal

## Abstract

The phenolic fraction of the extra virgin olive oil (EVOO) has been studied over the past two decades because of its important health protective properties. Numerous studies have been performed in order to clarify the most crucial factors that affect the concentration of the EVOO’s phenolic fraction and many contradictory results have been reported. Having as target to maximize the phenolic content of EVOO and its healthy properties we investigated the impact of harvest time, malaxation temperature, and malaxation duration on the concentration of individual phenols in extra virgin olive oil. Olive oil was prepared in a lab-scale olive mill from different varieties in Greece. The extraction process for cultivar (cv) Koroneiki samples was performed at five different harvest periods from the same trees with three different malaxation temperatures and five different malaxation duration times (N = 75). Similar types of experiments were also performed for other varieties: cv Athenolia (N = 20), cv Olympia (N = 3), cv Kalamata (N = 3), and cv Throubolia Aegean (N=3) in order to compare the changes in the phenolic profile during malaxation. The quantitative analysis of the olive oil samples with NMR showed that the total phenolic content has a negative correlation with the ripening degree and the malaxation time. The NMR data we collected helped us to quantitate not only the total phenolic content but also the concentration of the major phenolic compounds such as oleocanthal, oleacein, oleokoronal, and oleomissional. We noticed different trends for the concentration of these phenols during malaxation process and for different malaxation temperatures. The different trends of the concentration of the individual phenols during malaxation and the completely different behavior of each variety revealed possible biosynthetic formation steps for oleocanthal and oleacein and may explain the discrepancies reported from previous studies.

## 1. Introduction

The traditional Mediterranean diet, which is attracting continuous interest from the scientific community because of its health protecting properties, is based on the daily consumption of olive oil as the major source of lipids. Secoiridoid phenolic derivatives are one of the most important classes of minor constituents of olive oil, which present an increasing potential for health protection. The European Union (EU 432/2012) based on the scientific opinion of EFSA has permitted since 2012 a specific health claim related to the levels of phenolic compounds referred to as derivatives of hydroxytyrosol (**1**) found in olive oil [1].

Olive oil is a natural product, which is obtained directly from olive fruits and solely by mechanical means. The extraction is achieved by crushing the olive fruits and malaxing for a short time under mild heating. The separation of the olive paste and olive oil in modern olive mills is accomplished by centrifugation. The malaxation step is a prerequisite step in order to extract the maximum quantity of the olive oil from the olive paste. Malaxation favors coalescence of small oil droplets into larger systems which are easily separated from the aqueous phase through mechanical procedures, breaking up the oil/water emulsions formed during the crushing [2].

**Figure 1:**
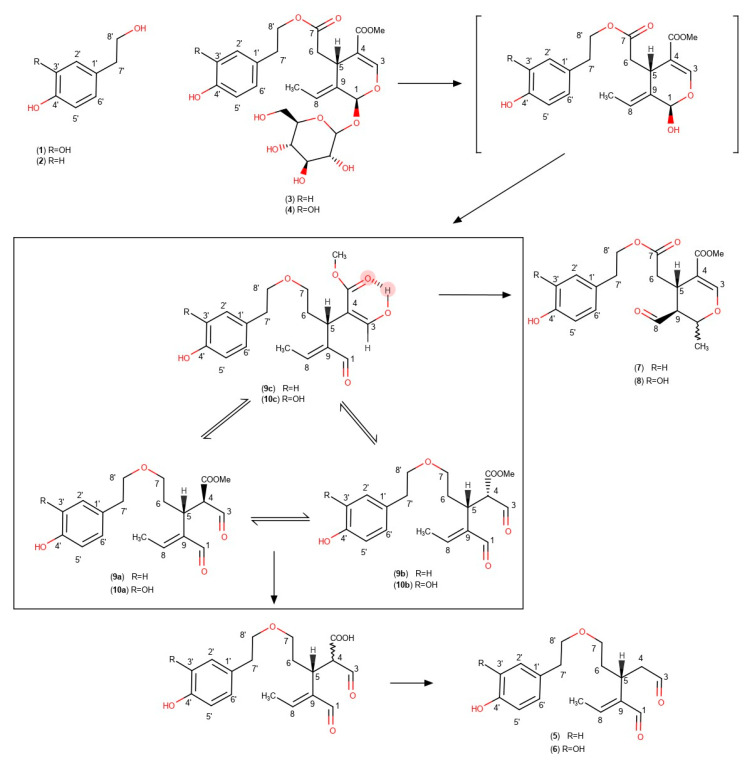
Proposed mechanism of oleocanthal (**5**) and oleacein (**6**) formation, starting from ligstroside (**3**) or oleuropein (**4**) through oleokoronal (**9**) or oleomissional (**10**). Hydroxytyrosol (**1**), tyrosol (**2**), ligstroside aglycon monoaldehydic form (**7**), and oleuropein aglycon monoaldehydic form (**8**) are also represented.

Olive oil phenols, which mainly include esterified derivatives of hydroxytyrosol (**1**) and tyrosol (**2**) (Figure 1), are not only responsible for the healthy properties of olive oil but also responsible for its shelf-life [3]. For this reason, many studies in the past have tried to investigate the factors that affect the phenolic content of olive oil. Contradictory results may be found in the literature, but most researchers agree that the crucial factors affecting the phenolic concentration of extra virgin olive oil (EVOO) are: (a) The genetic origin, (b) harvesting time, (c) malaxation temperature, and (d) malaxation duration.

The harvest period and consequently the maturity index of the fruit seem to be the most crucial agronomic factors that have been studied. Many researchers have reported that the concentration of phenols in olive oil decreases during ripening [4,5,6,7,8] while others present a nonlinear relation between olive ripeness and phenol concentration and especially, total phenolic content seems to increase in the early stages reaching a maximum concentration and after this point there is a decrease during ripening [9,10]. During olive ripening, chemical reactions and changes occur in the enzymatic activity, that induce variations in the chemical structure and concentration of some compounds of the olive fruit like ligstroside (**3**) and oleuropein (**4**) [11]. Previous studies have also reported variation of enzymatic activity during ripening for different olive fruit varieties [12].

Heating of the olive paste is a common practice in order to facilitate the merging of the olive droplets and break the oil/water emulsions. The applied heating has been an object of several research works investigating its possible effects on olive oil characteristics. Some authors have reported the negative relation between malaxation temperature and the concentration of total phenols [13,14]. The researchers noted that the increase of malaxation temperature affects the partition coefficient of hydrophilic phenols between the oil and the water phase, as related to their solubility [15]. In addition, the increase of temperature enhances the activity of endogenous peroxidase (POD) and polyphenol oxidase (PPO) enzymes which catalyze oxidative reactions in the malaxation matrix [11]. Opposite results have been reported by several authors showing a positive relation between malaxation temperature and concentration of phenols [16,17,18]. The authors explain that additionally to POD and PPO enhanced activity, probably there is an enhancement of the activity of other important enzymes that either catalyze formation reactions during the malaxation step, or let larger amounts of polyphenols to be released from the cell wall [4,16]. There are also previous works that note a nonlinear, but bell-shape relation between the malaxation temperature and the concentration of polyphenols [19,20]. All those different physicochemical phenomena that take place during olive oil malaxation, affect not only the total phenolic content of olive oil, but also affect the composition of the phenolic fraction qualitatively. Some researchers have noted a positive relation between temperature and oleocanthal (**5**) and oleacein (**6**) [5,6,21] while oleuropein and ligstroside aglycons (**7, 8**) show nonlinear correlation. Some researchers also report a bell shape relation between temperature and the concentration of phenolic constituents, with a peak at 32 °C has been reported for all substances [20].

Another crucial factor seems to be the malaxation duration. Some authors have reported a weak relation between malaxation duration and total phenol concentration [2,6,17,18,21,22,23,24]. On the contrary, some researches show a significant negative relation between malaxation time and phenol concentration [7,13]. The authors support that the decrease of the phenolic concentration in the olive oil is caused either by hydrolytic and oxidative enzymes like PPO and POD, or due to the prolonged contact between the paste and the vegetation water, favoring the diffusion of phenols into the aqueous phase. According to some authors, oleocanthal (**5**) and oleacein (**6**) increase their concentration during malaxation while aglycons show a weaker dependence from malaxation duration [5,6]. Contradictory results are reported by other authors, which show that oleacein is negatively affected by the duration of malaxation process, while oleocanthal shows increased concentration after prolonged malaxation [21]. 

The enzymatic activity during the olive paste malaxation has a significant effect on the concentration of phenols. The complexity of the enzymatic reactions seems to be partially responsible for the observed discrepancies among the different studies. This complexity of the enzymatic reactions among other factors is related with the strong dependence of the enzyme activity from the matrix temperature and from the malaxation duration. The maximum enzymatic activity of PPO is reported at high temperature (40–50 °C) but with very low stability in time whereas POD, has a weaker dependence on temperature [16]. During malaxation POD seems to have the strongest activity at 40 min of malaxation while PPO was not affected by the time [22]. Apart from the enzymatic activity, the solubility of phenols between the oil and the water phase has a significant effect and is also affected by the temperature and the duration of malaxation [21]. Considering also that each phenolic constituent is affected in a different way from all those factors makes it difficult to propose a simple trend for the fate of each phenol during malaxation. 

The study of the behavior of individual phenolic components during malaxation can reveal important steps of the biosynthetic pathway of important phenols like oleocanthal and oleacein. In the past years many researchers have proposed biochemical mechanisms of secoiridoids derivatives formation based on the data from the analysis of olive oil phenolic profile [11]. Recently, new forms of oleuropein and ligstroside aglycons named oleokoronal (**9**) and oleomissional (**10**) were presented after the analysis of olive oil with NMR [25]. In the same research, the authors highlighted the transformation of these forms of aglycons to other isomeric forms, after interaction of olive oil extracts with silica-based stationary phases. NMR analysis of olive oil phenolic fraction is a method with a lot of advantages in comparison to chromatographic methods offering direct, fast, and precise results and avoiding the formation of artefacts and the need of standards [26,27,28,29]. NMR analysis is even appliable in experiments with no extraction step that offers accurate information not only for the lipid fraction but also for the phenolic fraction via 1D and 2D experiments [30,31,32,33]. The use of inappropriate chromatographic methods in previous relative studies may be partially responsible for misleading results.

Having as target to maximize the phenolic content of EVOO and consequently its health-protecting properties, we investigated for the first time using qNMR, the impact of harvest time, malaxation temperature, and malaxation time on the concentration of individual phenols in extra virgin olive oil. In the current study, we investigated extensively the effect of all the above factors on the phenolic profile of cv Koroneiki from Greece, during the harvest season 2017–2018. We also investigated the effect on cv Athenolia and finally we compared the changes happening to the phenolic substances during malaxation in three more varieties (Olympia, Kalamata, and Throubolia Aegean) for the harvest seasons 2017–2018 and 2018–2019.

## 2. Results and Discussions

### 2.1. Impact of Olive Fruit Ripening 

**Figure 2:**
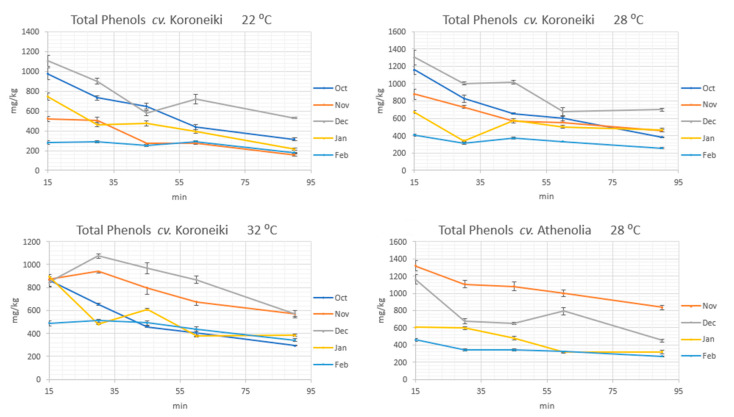
The total phenolic concentration of cv Koroneiki at five different ripening degrees during malaxation time at three different temperatures and for cv Athenolia at 28 °C, at four different ripening degrees.

For the current study we prepared 95 olive oil samples, using olive fruits from the same trees of cv Koroneiki (N = 75) and cv Athenolia (N = 20) collected at different ripening degrees. The analysis of the phenolic content of each olive oil sample (Figure 2 and Tables S1–S6) showed that the ripening degree had a significant effect on the total phenolic content of olive oil. For both varieties, the total phenolic content was decreasing during ripening. To avoid misleading results due to the impact of malaxation time, we compared the effects of the ripening degree at every time-point of malaxation during the entire process of malaxation and focused on the early steps of malaxation (15 min) when the effect was more intense. For the cv Koroneiki the decrease was observed in every studied malaxation temperature. In fact, in the early ripening stage (October) there is a high concentration of the total phenolic content. This content seems slightly to increase in the next months of the early ripening (November and early December) and after this point, the total phenolic content seems to decrease. Additionally, the reduction of the phenolic content becomes more intense as we get closer to higher ripening degrees (Figure 2). However, it should be noted that depending on the climatic conditions every year the same ripening stage (not accurately related with the skin color) may be observed even one month earlier, meaning that the studied harvest time points have a relative value. 

During ripening many enzymatic transformations take place which are mainly responsible for the reduction of the phenolic concentration. During ripening also, there are many changes of the composition of the olive fruit and as a result, the physicochemical matrix inside the malaxer, after olive fruit crush, is significantly different at each ripening stage.

### 2.2. Impact of Malaxation Temperature

The impact of malaxation temperature was mainly studied in cv Koroneiki. In these experiments we observed that the malaxation temperature had also a significant effect on the total phenolic content. In most of the experiments, when malaxation temperature increased from 22 °C to 28 °C or 32 °C the total phenolic content also increased its maximum concentration. The increase of the total phenolic content, depending on the malaxation duration, reached up to 40% (300 mg/kg to 500 mg/kg) at the latest ripening stage (Figure 1).

**Figure 3:**
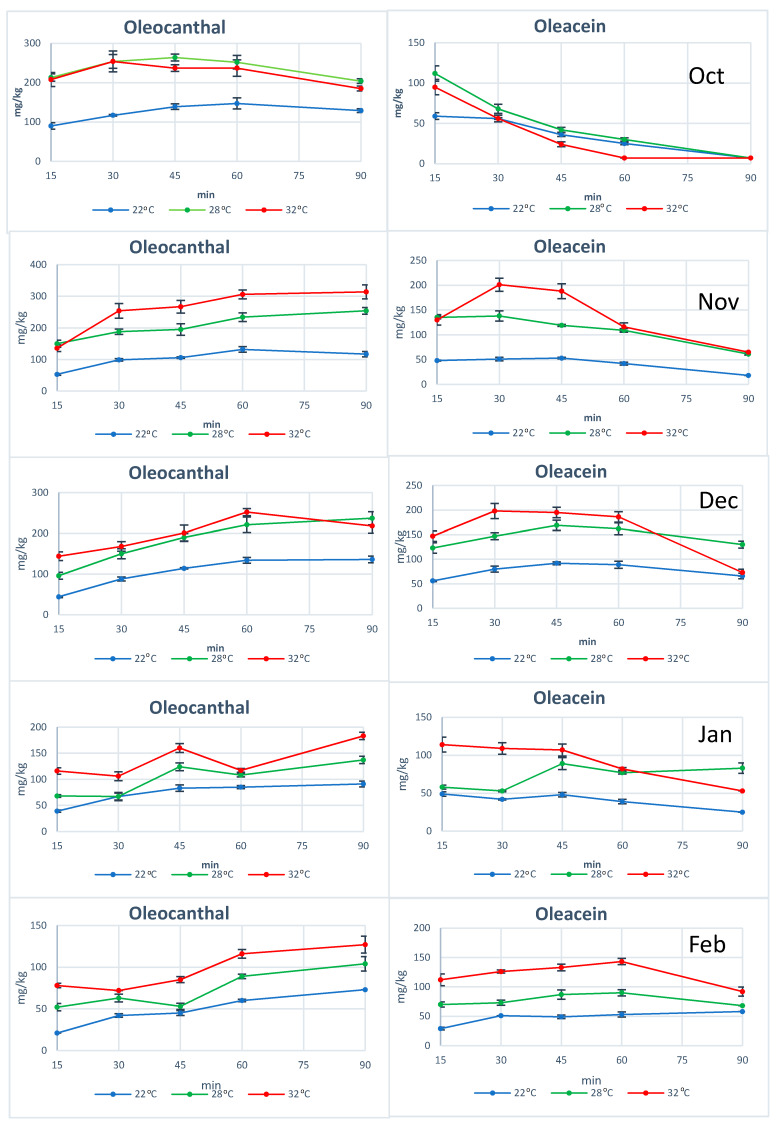
The concentration of oleocanthal and oleacein at five different ripening degrees during malaxation at three different temperatures.

**Figure 4:**
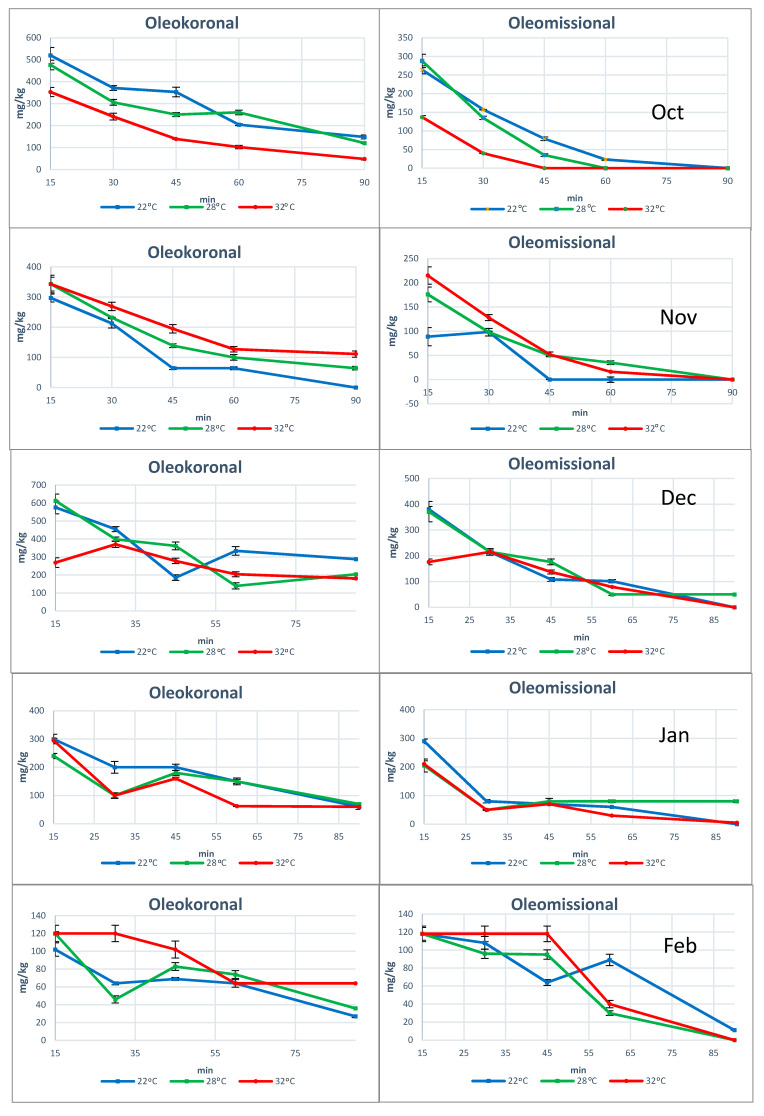
The concentration of Oleokoronal and Oleomissional at five different ripening degrees during malaxation at three different temperatures.

Concerning the individual phenols, in all the experiments we noticed that both oleocanthal and oleacein increased their concentration with the increase of temperature (Figure 3). When we examined the concentration of these two phenols in the early stages of malaxation (15–45 min) we noticed that in every ripening degree, the concentration achieved at 28 °C or 32 °C was higher than in the low temperature 22 °C (Figure 3) with statistically significant difference (*p* < 0.05) (Tables S12 and S13). On the contrary, oleokoronal and oleomissional showed the opposite trend but with a weaker tense. In the early stages of malaxation (15 min) oleokoronal and oleomissional had lower concentration at higher temperature (Figure 4). As many researchers have already reported, the concentration of phenols is affected by many factors. The most important of them is the enzymatic activity and the solubility of the phenols in the vegetable waters. The impact of temperature on these phenols showed that at higher temperatures, the enzymatic activity favors the formation of oleocanthal and oleacein and not of oleokoronal and oleomissional. Considering that the solubility of oleokoronal and oleomissional on the one hand and oleocanthal and oleacein on the other hand, do not have significant differences, it seems that the main cause of this different trend in these two groups is not related with the partition between the oil and the aqueous phase.

### 2.3. Impact of Malaxation Duration

The malaxation duration had also a negative statistically significant effect on the concentration of the phenols in the olive oils prepared from cv Koroneiki and cv Athenolia (Table S11). In all the experiments we performed with cv Koroneiki at low (22 °C) and medium temperature (28 °C) the maximum concentration of the total phenols appeared at 15 min of malaxation. After this time point of malaxation, the total phenolic content decreased (Figure 2). Concerning the higher temperature (32 °C), the maximum concentration of the total phenols appeared at 30 min of malaxation at only two ripening degrees. The same results were also obtained from cv Athenolia where only the medium temperature was studied (Table S6).

**Figure 5:**
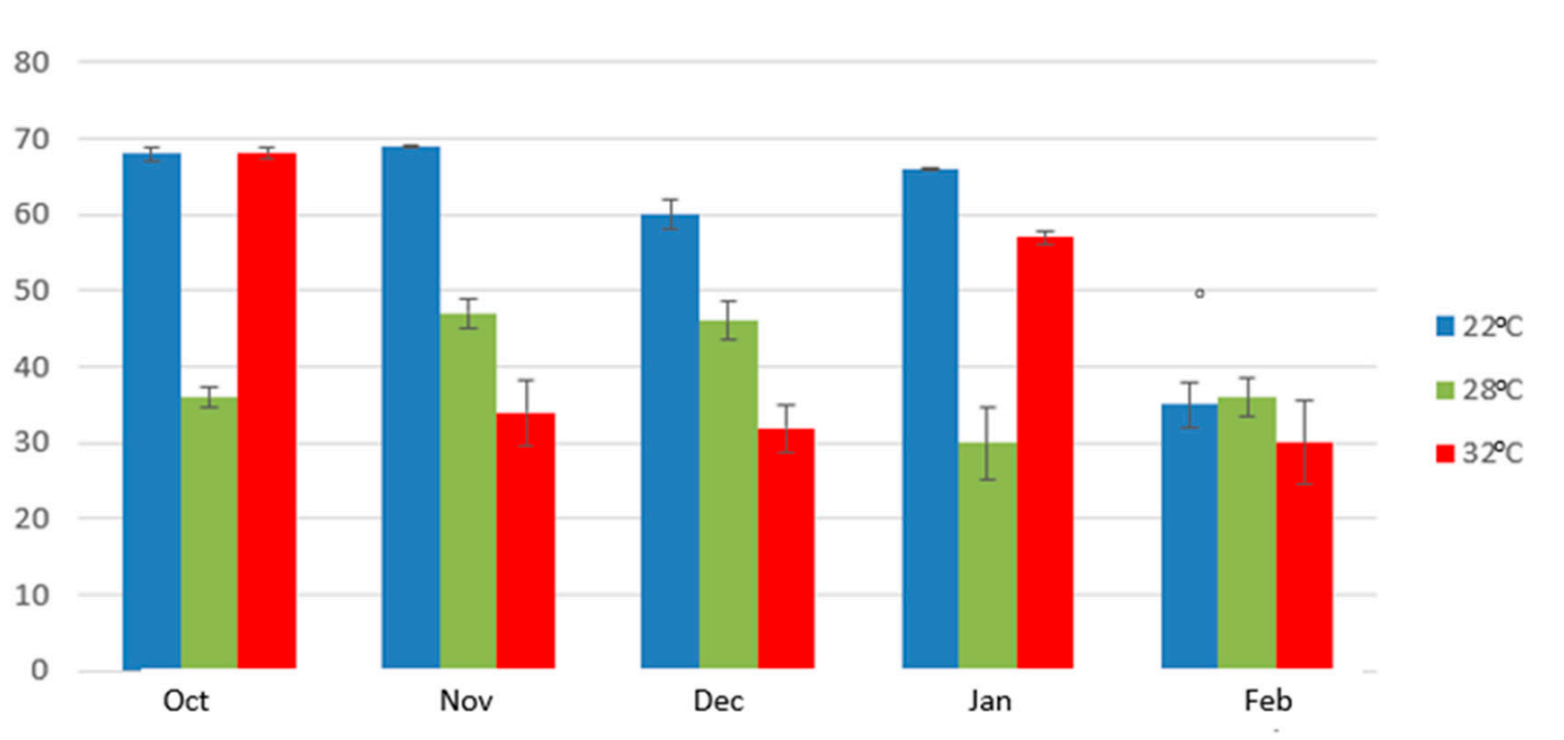
The % decrease of phenolic substances from 15 min to 90 min of malaxation for cv Koroneiki at three malaxation temperatures.

Many authors agree with these results, mentioning that the activity of the hydrolytic and oxidative enzymes and additionally the diffusion of the phenols to the vegetable waters decrease the concentration of phenols along with the time of malaxation [7,13]. The impact of the malaxation time on the decrease of the total phenolic content seems to be more intense at the early stages of ripening. The maximum decrease reached 70% (from 1160 mg/kg to 364 mg/kg) between 15 min and 90 min of malaxation at 20 November. As the olive fruit is ripening the impact of the malaxation time on the loss of phenolic substances becomes weaker (Figure 5).

The most interesting results were obtained from the investigation of the individual phenols during malaxation. In all ripening degrees of cv Koroneiki, we noticed that when we applied low temperature, the concentration of oleocanthal was increasing at least until 45 min of malaxation. In one case, the increase lasted until the 60th min. Concerning the concentration of oleacein we noticed that the increase of its concentration lasted until the 30th min of malaxation. In some cases, the increase stopped at 45 min. When higher temperature was applied, we noticed statistically significant higher levels of oleocanthal and oleacein concentration as we mentioned before (Tables S12 and S13). Additionally, we noticed that the concentration of oleocanthal had an increasing trend in the higher temperatures. Respectively, oleacein showed an increasing trend during malaxation which lasted until the 30th min in 4 out of 5 harvest time points (Figure 3). Especially for oleacein which is an orthodiphenolic compound and is more susceptible to enzymatic and chemical oxidation, it should be noted that as discussed in 2.5, oleacein is produced from oleomissional during malaxation (through demethylation and decarboxylation) and this can overcome the loss due to chemical and enzymatic oxidation.

Opposite results were obtained for the second group of studied phenolic substances. Both oleokoronal and oleomissional showed the maximum of their concentration at the very early stages of the malaxation process. Both oleomissional and oleokoronal concentration showed a decreasing trend during malaxation. There were only two cases where oleokoronal and oleomissional increased until the 30th min of malaxation. These two cases coincide with the experiments where the total phenolic content increased until the 30th min malaxation (Figure 4).

### 2.4. Changes of the Phenolic Profile During Malaxation in Four Different Cultivars

**Figure 6:**
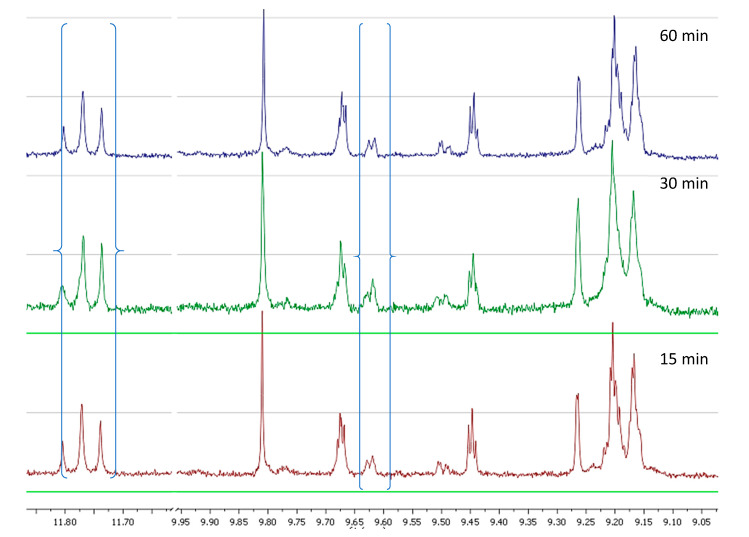
NMR profile of cv Throubolia Aegean at 28 °C, at three malaxation times, showing the characteristic peaks of the analyzed phenolic compounds.

In the second set of experiments we prepared olive oil from four different cultivars under exactly the same conditions and the same stage of ripening and investigated the changes happening during the malaxation. All results are presented in supplementary Tables S7 and S10. From the spectra of the cv Throubolia Aegean we observed that the phenolic profile remained almost the same during the 60 min of malaxation, in contrast to what we had observed for cv Koroneiki and Athenolia. Oleokoronal and oleomissional were the major constituents of the phenolic fraction, while oleocanthal and oleacein had a very low concentration. The only change we noticed during malaxation was a weak decrease of oleokoronal and oleomissional probably because of the PPO activity in the olive paste matrix (Figure 6).

**Figure 7:**
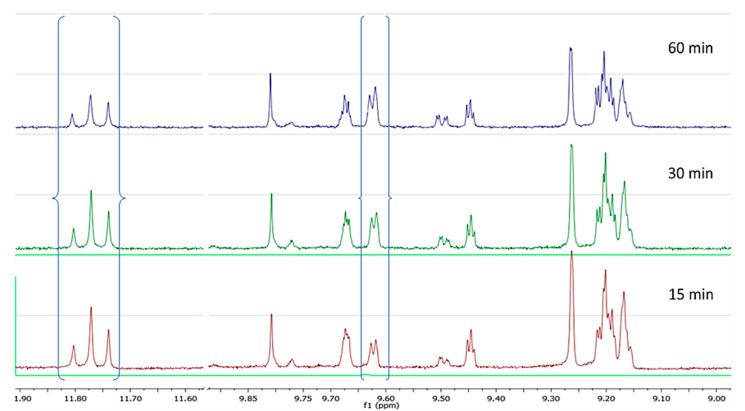
NMR profile of cv Olympia at 28 °C, at three malaxation times, showing the characteristic peaks of the analyzed phenolic compounds.

From the spectra of cv Olympia we observed that in the early stages of malaxation the major constituents were again oleokoronal and oleomissional while oleocanthal and oleacein were at low concentration. As the malaxation time was increasing, there was a weak decrease of oleokoronal and oleomissional peak and a weak increase of oleocanthal and oleacein peak. After 60 min of malaxation the major constituents were still oleokoronal and oleomissional (Figure 7).

**Figure 8:**
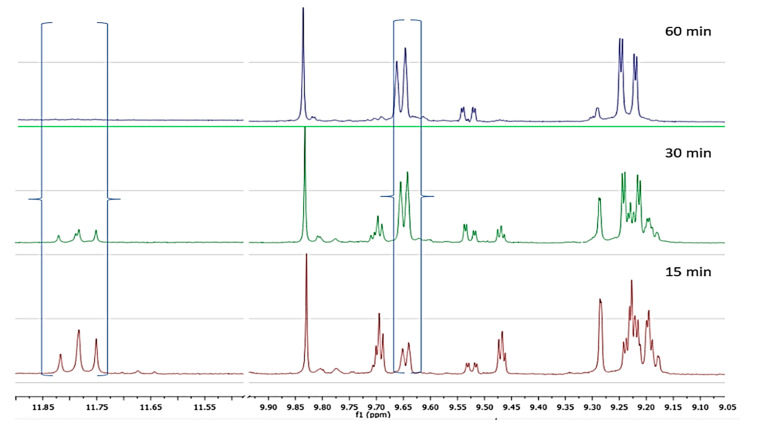
NMR profile of cv Koroneiki at 28 °C, at three malaxation times, showing the characteristic peaks of the analyzed phenolic compounds.

In the sample of cv Koroneiki variety, after 15 min of malaxation the major constituents were oleokoronal and oleomissional although this time oleocanthal and oleacein showed much higher concentration compared to the previous two cases. As the malaxation time was increasing we noticed that the concentration of oleokoronal and oleomissional was decreasing and at the same time the concentration of oleocanthal and oleacein was increasing. After 60 min of malaxation most of the oleokoronal and oleomissional had disappeared (Figure 8).

**Figure 9:**
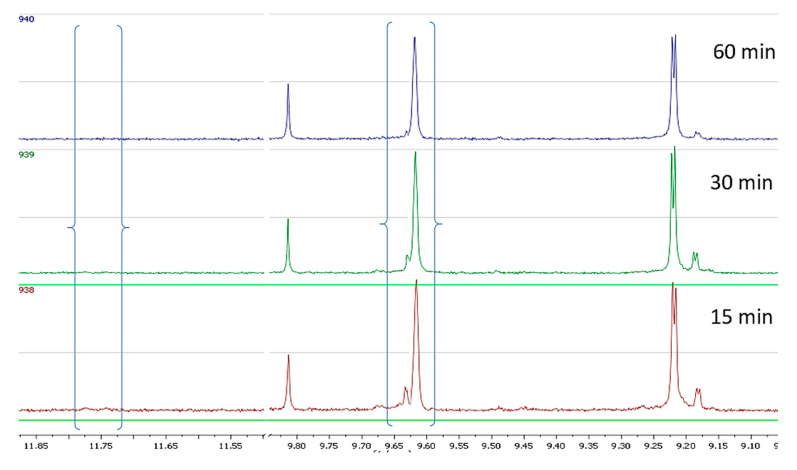
NMR profile of cv Kalamata at 28 °C, at three malaxation times, showing the characteristic peaks of the analyzed phenolic compounds.

In the last experiment with cv Kalamata we noticed that by the 15th min of malaxation there was total absence of oleokoronal and oleomissional or any other form of oleuropein and ligstroside aglycons while the maximum concentration of oleocanthal and oleacein had already been achieved. In the next stages of malaxation the only observed change was the degradation of oleocanthal and oleacein due to PPO activity (Figure 9).

### 2.5. Proposed Mechanism for Phenolic Ingredients Transformations During Malaxation

In a recent study, we presented a number of new forms of oleuropein and ligstroside aglycons, thoroughly investigated with the NMR spectroscopy [25]. The changes happening during malaxation for each individual phenol, reveal some important clues which are related with the mechanism of the formation of oleocanthal and oleacein. The main metabolites that are found in the intact and unripe olive fruit are oleuropein and ligstroside. These two metabolites are glucosidic derivatives of elenolic acid esterified with hydroxytyrosol or tyrosol. However, the phenolic constituents found in olive oil, with the exception of free tyrosol and hydroxytyrosol, are not present in the intact olive fruit or are present at very low concentrations. Obviously, all these constituents including oleocanthal, oleacein and all the forms of oleuropein and ligstroside aglycons are the result of multiple enzymatic reactions happening during crushing and malaxation. 

In previous studies, it has been mentioned that a few seconds after the olive fruit crush the main phenolic constituent in the olive paste is oleuropein [21]. After some minutes of malaxation the concentration of oleuropein is significantly decreasing followed by increase of oleuropein aglycons. The researchers, contrary to our results, are referring the aglycons **7** and **8**. As we have mentioned in a previous work [25], these forms of oleuropein aglycon and ligstroside aglycon are produced at least at some extent by the reaction between oleokoronal and oleomissional with silica gel showing artificially higher concentrations. Additionally, these two forms are stable and cannot be further transformed to other forms like oleocanthal and oleacein. As we observed in the experiments with cv Koroneiki or Athenolia during the early steps of the malaxation procedure, the major constituents of olive oil are oleokoronal and oleomissional, while oleocanthal and oleacein have always lower concentration. As the malaxation procedure continues, large amounts of oleokoronal and oleomissional disappear followed by a simultaneous increase of oleocanthal and oleacein while oleuropein and ligstroside aglycons (**7**, **8**) do not present significant changes. This result led as to the conclusion that oleocanthal comes from the enzymatic transformation of oleokoronal and oleacein from the transformation of oleomissional.

This conclusion is supported by the fact that the transformation proceeding is affected by the malaxation temperature. Comparing olive oil produced from the same olive fruits in three different malaxation temperatures for 15 min we noticed that the increase of temperature at 28 °C and 32 °C seems to enhance the transformation of oleokoronal to oleocanthal. Contrariwise, at low temperature (22 °C) the phenolic profile is very different: oleocanthal is at least 50% lower while oleokoronal and oleomissional are found in higher concentrations.

Obviously, the yield of this transformation is strongly affected by the malaxation conditions. It is also apparent that the yield of this transformation is different from variety to variety and this can explain the discrepancies in the literature about the impact of malaxation on the phenolic content of olive oil. In the experiments we performed, we noticed that there are some varieties like cv Kalamata in which there is a rapid and total transformation of oleokoronal and oleomissional to oleocanthal and oleacein, just after 15 or 30 min of malaxation. Other varieties, like cv Koroneiki, follow the same biochemical pathway but more slowly (60 or 90 min). In varieties like cv Olympia we noticed that the above-mentioned transformations have not been completed even after 90 min of malaxation. Surprisingly, there are some varieties like Throubolia Aegean from the island of Naxos, in which there is total absence of this transformation. After 60 min of malaxation only extremely small quantities of oleocanthal and oleacein have been formed, indicating the total absence (or inactivity) of any transforming enzymes apart from β-glucosidase (Figure 6). In a recent paper, the presence of methylesterases in the olive fruit was mentioned for the first time confirming the assumption of existence of “transforming enzymes” [34]. Volk et al. have shown that successive action of β-glucosidase and methyl esterase to oleuropein and ligstroside leads to oleacein and oleocanthal respectively. However, they have not shown experimentally which exact forms of the intermediate aglycons are implicated. We show here, in real samples, that during malaxation the concentration of oleomissional and oleokoronal decreases while that of oleacein and oleocanthal increases indicating that those forms are the mainly implicated intermediates and not the monoaldehydic cyclic forms of the aglycons (**7,8**). Considering all these data, we can propose that the biosynthetic pathway of oleocanthal and oleacein, includes a first step of hydrolysis of the glucosidic bond of ligstroside and oleuropein catalyzed by β-glucosidase, leading to oleokoronal and oleomissional and a second step of removal of the methyl-group catalyzed by a methylesterase. The produced carboxylic derivatives are unstable forms that can be easily thermally decarboxylated leading to oleocanthal and oleacein (Figure 1).

## 3. Materials and Methods

### 3.1. Chemicals and Standards

All solvents were of analytical grade (Merck). Syringaldehyde (98% purity, Sigma–Aldrich, Steinem, Germany) was used as internal standard (IS). IS solution was prepared in acetonitrile at a concentration of 0.5 mg/mL and kept at 4 °C. Prior to use the IS solution was left to come to room temperature.

### 3.2. Instrumentation

The quantitative determination of phenolics in olive oil was performed using NMR spectroscopy with a Bruker Avance 400 MHz (University of Athens). The ^1^H-NMR spectra were recorded for each sample in CDCl_3_ and processed using either the MNova (Mestrelab Research) or the TOPSPIN software.

For the extraction process we used an olive crusher, electronic thermometer, a stainless steel malaxer and a centrifuge device. All evaporations were performed at 40 °C using rotary evaporator (Buchi, Flawil, Switzerland).

### 3.3. Sampling of Olive Fruits and Olive Oil Preparation Process

At the first set of experiments, olive fruits of the cv Koroneiki were collected by hand every month from the area of Molaoi, Greece, at five different ripening degrees, from 10 October 2017 until 10 February 2018 from a non-irrigated orchard. For each sampling we used every time a mixture of olive fruits coming from exactly the same three adjacent trees of the same age at equal quantity from each tree (1000 g from each tree). The fruits were pooled to one sample to avoid variability from tree to tree, and was then randomly divided to three parts that were further used for the experiments studying the effect of malaxation temperature and duration (each malaxation experiment at each harvest time was repeated three times). Olive fruits of the cv Athenolia were collected from three trees, from the same area from a non-irrigated orchard, at four different ripening degrees from 10 November 2017 until 10 February 2018 (experiments similarly performed in triplicate). In both cases, the olive fruits were stored at ambient temperature in open paper boxes and transferred within 24 h to the laboratory. A second series of experiments was performed with one sample of olive fruits (randomly divided to three parts) from cv Κalamata from the region of Sparta, Greece, cv Olympia (syn. Choraitiki) from the region of Olympia, Greece, cv Koroneiki from Kalamata, Greece and cv Throubolia Aegean from the island of Naxos, Greece. In the second set of experiments we used only 100% green olive fruits at the same early degree of ripening.

The lab scale olive mill consisted of a lab scale hammer crusher operating at 1500 rpm, a stainless steel semicylindrical malaxer with 3 L capacity, and a centrifuge device (FRONTIER™ 5000 SERIES MULTI OHAUS), able to simulate the olive oil preparation that takes place in an olive mill. The olive fruits were crushed (particle size < 7 mm) and within 5 min were transferred to the malaxer. The olive paste was malaxed under slow kneading for 90 min. Temperature control was achieved with warm water through the stainless-steel wall and monitored inside the olive paste with an electronic thermometer. For cv Koroneiki samples, at each ripening degree, we prepared the olive oil at three different temperatures: 22 °C, 28 °C, and 32 °C and we collected olive oil samples at five different malaxation times (15, 30, 45, 60, 90 min) (N = 75). For the cv Athenolia, at each ripening degree we prepared the olive oil only at 28 °C and we collected olive oil samples at five different malaxation times (15, 30, 45, 60, 90 min) (N = 20). At each time point, 200 g of the olive paste were transferred in a plastic tube, and centrifuged for 5 min at 4000 rpm. The olive oil phase was collected, weighed, and immediately analyzed as described below. For the second set of experiments only one extraction was performed for each sample, applying 28 °C malaxation temperature and samples for analysis were collected at 15, 30, and 60 min (N = 3 for each variety). More detailed description of sampling and planning of the experiments is presented in Supplementary Table S14.

### 3.4. Olive Oil Extraction for Analysis

For the extraction of the phenolic fraction from the olive oil samples we followed the protocol of Karkoula [28] et al. Olive oil (5.0 g) was mixed with cyclohexane (20 mL) and acetonitrile (25 mL). The mixture was homogenized using a vortex mixer (VXMTAL multi-tube vortex mixer, OHAUS) for 60 s and centrifuged at 4000 rpm for 5 min. A part of the acetonitrile phase (25 mL) was collected, mixed with 1.0 mL of a syringaldehyde solution (0.5 mg/mL) in acetonitrile, and evaporated under vacuum using a rotary evaporator.

### 3.5. NMR Spectra Analysis

The residue of the above procedure was dissolved in CDCl_3_ (750 μL) and an accurately measured volume of the solution (550 μL) was transferred to a 5 mm NMR tube. ^1^H-NMR spectra were recorded at 400 MHz (Bruker DRX400). Typically, 32 scans were collected into 32K data points over a spectral width of 0−16 ppm with a relaxation delay of 1 s and an acquisition time of 1.7 s. Prior to Fourier transformation (FT), an exponential weighting factor corresponding to a line broadening of 0.3 Hz was applied. The spectra were phase corrected and the baseline was fixed manually.

### 3.6. Quantitation of Phenolic Constituents

**Figure 10:**
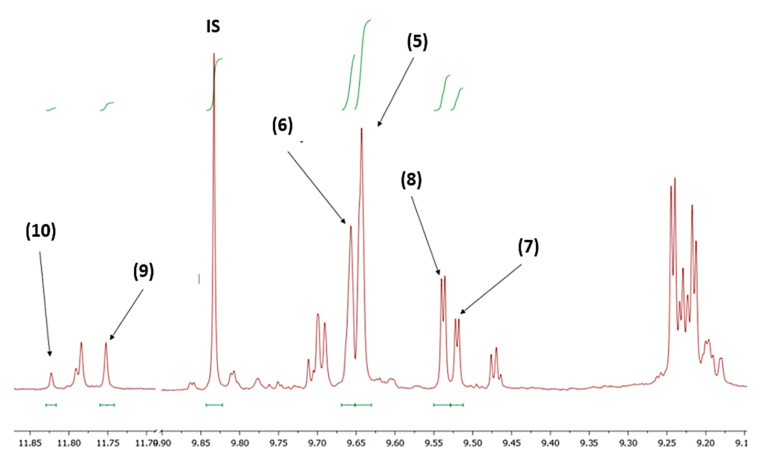
Example of NMR spectrum (400 MHz) of the aldehydic region of a typical olive oil extract depicting the selected peaks that were used for integration: oleocanthal (**5**), oleacein (**6**), ligstroside aglycon monoaldehydic form (**7**), oleuropein aglycon monoaldehydic form (**8**) oleokoronal (**9**), oleomissional (**10**). IS: internal standard (syringaldehyde).

For the analysis of the NMR data and the quantitation of the phenolic constituents we followed the protocol published by Karkoula et al. [28,29] with some ameliorations and modifications to include oleokoronal and oleomissional. For simplicity, the term Oleokoronal refers to a mixture of the two dialdehydic (**9a**, **9b**) and the enolic form (**9c**) of ligstroside aglycon which exist in olive oil in equilibrium at a stable ratio 1:1:2 respectively when the spectrum is recorded in CDCl_3_ at 298 K [25]. Similarly, Oleomissional refers to the mixture of the three forms of oleuropein aglycon (**10a**, **10b**, **10c**) which also exist in equilibrium at a ratio 1:1:2 respectively. For the peaks of interest, accurate integration was performed manually (Figure 10). Oleocanthal (**5**) concentration (C_1_) was measured using the relative integration of the aldehydic proton at 9.62 ppm (I_1_) and calculated using the equation C_1_ = 197.72 × I_1_ + 19.77. Oleacein (**6**) was calculated using the integration of the aldehydic proton at 9.64 ppm and the equation C_2_ = 205.09 × I_2_ + 19.16. Oleokoronal (**9**) was calculated using the integration of the enolic proton at 11.74 ppm and the equation C_3_ = 4 × (232.7 × I_3_ + 4.3) and ligstroside aglycon monoaldehydic form (**7**) by the aldehydic proton at 9.48 ppm and the equation C_4_ = 232.7 × I_4_ + 4.3. Oleomissional (**10**) was calculated using the integration of the enolic proton at 11.80 ppm and the equation C_5_ = 4 × (243.5 × I_5_ + 4.57) and oleuropein aglycon monoaldehydic form (**8**) using the integration of the aldehydic proton at 9.50 ppm and the equation C_6_ = 243.5 × I_6_ + 4.57. All results are expressed in mg/kg. The total phenol concentration C_tot_ = C_1_ + C_2_ + C_3_ + C_4_ + C_5_ + C_6_. All equations were based on calibration curves constructed using pure standards isolated from olive oil and the integration of the aldehydic proton of the internal standard at 9.81 ppm is set at 1. All compounds presented inter-day and intra-day precision RSD < 10% and LOQ = 5 mg/Kg.

### 3.7. Statistical Analysis

Analysis of variance (ANOVA), as well as Duncan’s multiple range test (MRT), based on the 0.05 level of significance, were performed with the SPSS v.20 software for Windows (IBM SPSS Statistics 2011, IBM Corp. New York, NY, USA).

### 3.8. Linear Regression

A simple linear regression model i.e., total phenols = a + b × (malaxation time), was applied to determine whether total phenol content was statistically significantly dependent on malaxation time in every treated temperature.

### 3.9. ANOVA

To determine whether any of the differences among the means of oleocanthal as well as among oleacein concentrations were statistically significant at the studied levels of malaxation temperature, i.e., 22 °C, 28 °C, and 32 °C, in the first three malaxation times of 15, 30, and 45 min, analysis of variance was performed at *p* = 0.05. Duncan’s multiple range test (MRT) was used for multiple comparisons (*p* = 0.05).

## 4. Conclusions

In the current study we investigated the impact of the ripening degree, malaxation temperature, and malaxation duration on the phenolic fraction of the extra virgin olive oil. Our research was mainly focused on cv Koroneiki and cv Athenolia and in both cases the results showed a significant negative correlation between the ripening degree and the total phenolic concentration. The same negative trend was observed for the correlation of the phenolic fraction with the malaxation duration while a positive correlation was observed with the malaxation temperature. The comparison among the five studied major Greek varieties revealed that in all cases the total phenolic content is negatively correlated with the malaxation duration but most interestingly each variety has its own specific behavior concerning the transformation of oleokoronal and oleomissional to oleocanthal and oleacein during the malaxation. This observation led us to clarify the formation steps of oleocanthal and oleacein which can explain the reported discrepancies in previous studies and opens a new field for the search of varieties able to produce higher amounts of specific phenols with increased healthy properties.

## Supplementary Materials

The following are available online. Figure S1: The concentration of Total Phenols in three different malaxation temperatures (22,28,32 °C) for five different harvest months, Table S1–S5: Quantitation data for oleocanthal, oleacein, oleomissional, oleokoronal and total phenols in Koroneiki variety in five different harvest months, three different malaxation temperatures and five different malaxation times, Table S6: Quantitation data for cv. Athenolia in four different harvest months, five different malaxation times at 28 °C, Table S7–S10: Quantitation data for cv Throubolia, cv Kalamata, cv Olympia, cv Koroneiki in three different malaxation times at 28 °C, Table S11: The regression b coefficients and their statistically significance levels as R^2^ of the linear regression model Total phenols = a + b×(malaxation time), Table S12 and S13: The results of the analysis of variance (ANOVA) and Duncan’s MRT at p = 0.05 for oleocanthal and oleacein, Table S14: Sampling and experimental planning.
