# Supplementary material for: Influence of Harvest Time and Malaxation Conditions on the Concentration of Individual Phenols in Extra Virgin Olive Oil Related to Its Healthy Properties
Source: Molecules. 2020 May 24;25(10):2449. doi: 10.3390/molecules25102449 (PMC7287853; doi:10.3390/molecules25102449)
Supplement: Supplementary file 1 [file molecules-25-02449-s001.pdf]

Supplementary data

Influence of harvest time and malaxation conditions on the concentration of individual phenols in extra virgin olive oil related to its healthy properties

Panagiotis Diamantakos, Triada Giannara, Maria Skarkou, Eleni Melliou, Prokopios Magiatis*

Laboratory of Pharmacognosy and Natural Products Chemistry, Department of Pharmacy, National and Kapodistrian University of Athens, Panepistimiopolis Zografou, Athens 15771, Greece

* Correspondence: magiatis@pharm.uoa.gr

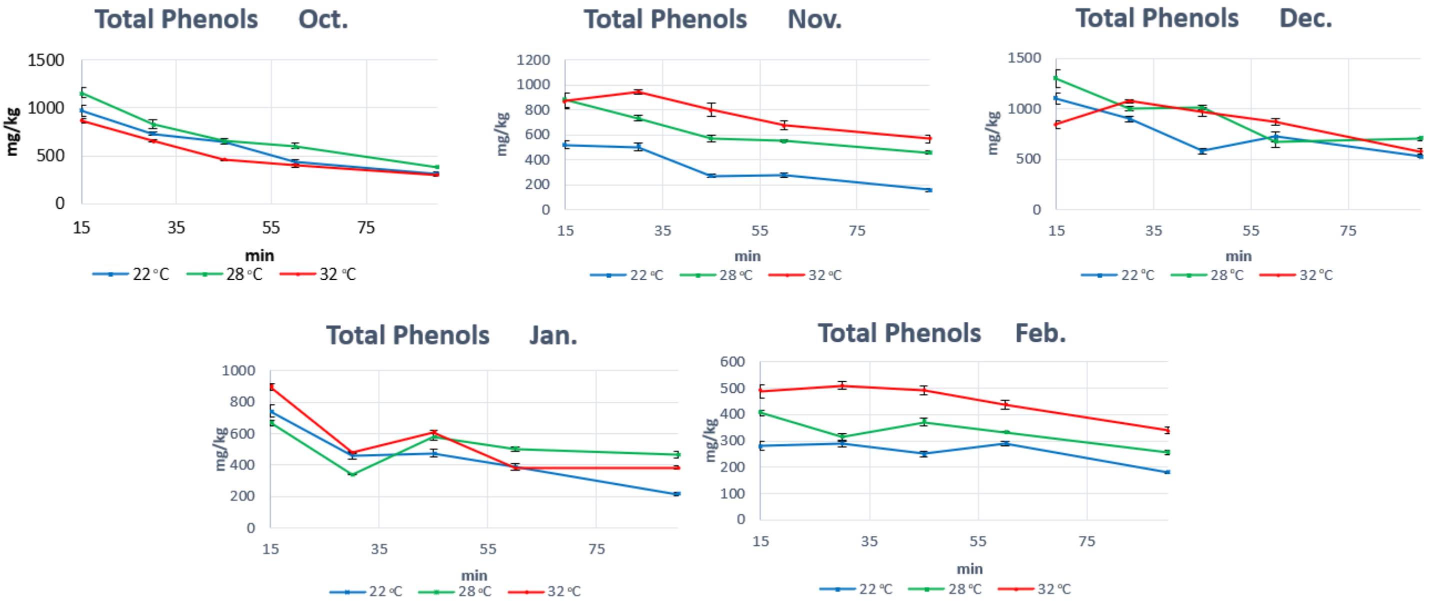

Figure S1. The concentration of Total Phenols in three different malaxation temperatures (22,28,32 °C) for 5 different harvest months

Table S1. Quantitation data for Oleocanthal in Koroneiki variety in 5 different harvest months, 3 different malaxation temperatures and 5 different malaxation times.

		cv. Koroneiki					
		T=22 ^{0}C		T=28 ^{0}C		T=32 ^{0}C	
**October**	Malaxation time	Oleocanthal mean	SD	Oleocanthal mean	SD	Oleocanthal mean	SD
	min	mg/Kg	mg/Kg	mg/Kg	mg/Kg	mg/Kg	mg/Kg
	15	90	8	213	9	208	18
	30	117	2	254	26	254	18
	45	139	7	264	9	237	9
	60	147	14	252	17	237	21
	90	129	5	204	6	185	6
**November**	Malaxation time	Oleocanthal mean	SD	Oleocanthal mean	SD	Oleocanthal mean	SD
	min	mg/Kg	mg/Kg	mg/Kg	mg/Kg	mg/Kg	mg/Kg
	15	53	3	150	11	136	11
	30	99	4	188	9	254	23
	45	106	2	195	18	267	20
	60	132	8	234	14	306	14
	90	117	8	254	10	314	22
**December**	Malaxation time	Oleocanthal mean	SD	Oleocanthal mean	SD	Oleocanthal mean	SD
	min	mg/Kg	mg/Kg	mg/Kg	mg/Kg	mg/Kg	mg/Kg
	15	44	2	96	9	144	11
	30	88	5	150	12	168	12
	45	114	2	190	10	201	20
	60	134	7	221	19	252	9
	90	136	8	237	16	218	18
**January**	Malaxation time	Oleocanthal mean	SD	Oleocanthal mean	SD	Oleocanthal mean	SD
	min	mg/Kg	mg/Kg	mg/Kg	mg/Kg	mg/Kg	mg/Kg
	15	39	2	68	3	116	6
	30	67	6	67	8	106	9
	45	83	6	124	8	160	9
	60	85	3	108	4	117	4
	90	91	6	137	7	183	7
**February**	Malaxation time	Oleocanthal mean	SD	Oleocanthal mean	SD	Oleocanthal mean	SD
	min	mg/Kg	mg/Kg	mg/Kg	mg/Kg	min	mg/Kg
	15	21	0	52	4	78	3
	30	42	2	63	5	72	0
	45	45	3	53	4	85	4
	60	60	1	89	3	116	5
	90	73	0	104	9	127	10

Table S2. Quantitation data for Oleacein in Koroneiki variety in 5 different harvest months, 3 different malaxation temperatures and 5 different malaxation times

		cv. Koroneiki					
		T=22 ^{0}C		T=28 ^{0}C		T=32 ^{0}C	
**October**	Malaxation time	Oleacein mean	SD	Oleacein mean	SD	Oleacein mean	SD
	min	mg/Kg	mg/Kg	mg/Kg	mg/Kg	mg/Kg	mg/Kg
	15	59	4	112	10	95	10
	30	56	4	68	6	56	1
	45	36	2	42	3	24	3
	60	25	2	30	2	7	0
	90	7	0	7	0	7	0
**November**	Malaxation time	Oleacein mean	SD	Oleacein mean	SD	Oleacein mean	SD
	min	mg/Kg	mg/Kg	mg/Kg	mg/Kg	mg/Kg	mg/Kg
	15	48	1	135	3	130	11
	30	51	4	138	10	201	13
	45	53	2	119	2	188	15
	60	42	3	109	3	116	8
	90	18	0	61	2	65	2
**December**	Malaxation time	Oleacein mean	SD	Oleacein mean	SD	Oleacein mean	SD
	min	mg/Kg	mg/Kg	mg/Kg	mg/Kg	mg/Kg	mg/Kg
	15	56	1	123	11	147	11
	30	80	6	147	7	198	16
	45	92	3	169	11	195	11
	60	89	7	162	12	186	11
	90	66	6	130	7	73	7
**January**	Malaxation time	Oleacein mean	SD	Oleacein mean	SD	Oleacein mean	SD
	min	mg/Kg	mg/Kg	mg/Kg	mg/Kg	mg/Kg	mg/Kg
	15	49	3	58	3	114	10
	30	42	1	53	1	109	8
	45	48	3	89	8	107	8
	60	39	3	77	2	82	2
	90	25	0	83	7	53	0
**February**	Malaxation time	Oleacein mean	SD	Oleacein mean	SD	Oleacein mean	SD
	min	mg/Kg	mg/Kg	mg/Kg	mg/Kg	min	mg/Kg
	15	29	2	70	4	112	10
	30	51	0	73	4	126	3
	45	49	3	87	8	133	6
	60	53	4	90	5	143	5
	90	58	0	68	0	92	8

Table S3. Quantitation data for Oleokoronal in Koroneiki variety in 5 different harvest months, 3 different malaxation temperatures and 5 different malaxation times

		**cv. Koroneiki**					
		T=22 ^{0}C		T=28 ^{0}C		T=32 ^{0}C	
**October**	Malaxation time	Oleokoronal mean	SD	Oleokorona l mean	SD	Oleokoronal mean	SD
	min	mg/Kg	mg/Kg	mg/Kg	mg/Kg	mg/Kg	mg/Kg
	15	520	36	476	22	353	21
	30	371	11	306	13	241	15
	45	353	22	250	9	139	2
	60	204	4	260	10	102	8
	90	148	9	120	2	48	4
**November**	Malaxation time	Oleokoronal mean	SD	Oleokorona l mean	SD	Oleokoronal mean	SD
	min	mg/Kg	mg/Kg	mg/Kg	mg/Kg	mg/Kg	mg/Kg
	15	297	13	343	29	343	23
	30	213	16	232	1	269	14
	45	64	5	139	6	195	14
	60	64	5	100	9	127	9
	90	n.d.	0	64	6	111	10
**December**	Malaxation time	Oleokoronal mean	SD	Oleokorona l mean	SD	Oleokoronal mean	SD
	min	mg/Kg	mg/Kg	mg/Kg	mg/Kg	mg/Kg	mg/Kg
	15	576	35	613	38	269	26
	30	455	14	399	12	371	18
	45	185	16	362	22	278	15
	60	334	24	139	17	204	14
	90	288	3	204	1	181	5
**January**	Malaxation time	Oleokoronal mean	SD	Oleokorona l mean	SD	Oleokoronal mean	SD
	min	mg/Kg	mg/Kg	mg/Kg	mg/Kg	mg/Kg	mg/Kg
	15	300	17	240	9	295	8
	30	200	21	100	9	100	10
	45	200	11	180	9	160	5
	60	150	12	150	7	62	1
	90	60	9	70	0	60	0
**February**	Malaxation time	Oleokoronal mean	SD	Oleokorona l mean	SD	Oleokoronal mean	SD
	min	mg/Kg	mg/Kg	mg/Kg	mg/Kg	min	mg/Kg
	15	102	7	120	2	120	9
	30	64	2	46	4	120	9
	45	69	2	83	4	102	10
	60	64	4	74	4	64	0
	90	27	1	36	0	64	0

Table S4. Quantitation data for Oleomissional in Koroneiki variety in 5 different harvest months, 3 different malaxation temperatures and 5 different malaxation times

		cv. Koroneiki					
		T=22 ºC		T=28 ºC		T=32 ºC	
**October**	Malaxation time	Oleomissional mean	SD	Oleomissional mean	SD	Oleomissional mean	SD
	min	mg/Kg	mg/Kg	mg/Kg	mg/Kg	mg/Kg	mg/Kg
	15	264	11	288	18	137	4
	30	157	1	135	4	40	1
	45	79	5	35	3	n.d.	0
	60	23	2	n.d.	0	n.d.	0
	90	n.d.	0	n.d.	0	n.d.	0
**November**	Malaxation time	Oleomissional mean	SD	Oleomissional mean	SD	Oleomissional mean	SD
	min	mg/Kg	mg/Kg	mg/Kg	mg/Kg	mg/Kg	mg/Kg
	15	89	19	176	15	215	18
	30	98	2	98	8	128	6
	45	n.d.	8	50	1	52	5
	60	n.d.	6	35	4	16	0
	90	n.d.	0	n.d.	0	n.d.	0
**December**	Malaxation time	Oleomissional mean	SD	Oleomissional mean	SD	Oleomissional mean	SD
	min	mg/Kg	mg/Kg	mg/Kg	mg/Kg	mg/Kg	mg/Kg
	15	381	11	371	40	176	11
	30	215	7	215	13	215	2
	45	108	7	176	11	137	8
	60	101	6	50	3	79	2
	90	n.d.	0	50	0	n.d.	0
**January**	Malaxation time	Oleomissional mean	SD	Oleomissional mean	SD	Oleomissional mean	SD
	min	mg/Kg	mg/Kg	mg/Kg	mg/Kg	mg/Kg	mg/Kg
	15	290	7	205	23	210	11
	30	80	6	50	1	50	2
	45	70	2	80	10	70	1
	60	60	1	80	5	30	1
	90	n.d.	0	80	5	5	0
**February**	Malaxation time	Oleomissional mean	SD	Oleomissional mean	SD	Oleomissional mean	SD
	min	mg/Kg	mg/Kg	mg/Kg	mg/Kg	min	mg/Kg
	15	118	7	118	9	118	9
	30	108	7	96	5	118	9
	45	64	3	95	5	118	9
	60	89	6	30	3	40	4
	90	11	0	n.d.	0	n.d.	0

Table S5. Quantitation data for Total Phenols in Koroneiki variety in 5 different harvest months, 3 different malaxation temperatures and 5 different malaxation times

	cv. Koroneiki						
		T=22 ⁰C		T=28 ⁰C		T=32 ⁰C	
**October**	Malaxation time	Total Phenols mean	SD	Total Phenols mean	SD	Total Phenols mean	SD
	min	mg/Kg	mg/Kg	mg/Kg	mg/Kg	mg/Kg	mg/Kg
	15	973	60	1320	60	867	25
	30	732	21	1106	40	654	11
	45	647	29	1081	51	457	6
	60	435	26	1000	35	406	29
	90	313	15	835	26	295	5
**November**	Malaxation time	Total Phenols mean	SD	Total Phenols mean	SD	Total Phenols mean	SD
	min	mg/Kg	mg/Kg	mg/Kg	mg/Kg	mg/Kg	mg/Kg
	15	520	29	878	60	871	59
	30	502	31	732	20	941	16
	45	271	10	569	27	798	55
	60	276	14	552	6	675	32
	90	157	10	457	16	568	31
**December**	Malaxation time	Total Phenols mean	SD	Total Phenols mean	SD	Total Phenols mean	SD
	min	mg/Kg	mg/Kg	mg/Kg	mg/Kg	mg/Kg	mg/Kg
	15	1102	56	1301	88	845	38
	30	901	28	1003	20	1075	19
	45	580	26	1015	22	969	49
	60	722	49	673	53	867	31
	90	528	6	700	16	574	28
**January**	Malaxation time	Total Phenols mean	SD	Total Phenols mean	SD	Total Phenols mean	SD
	min	mg/Kg	mg/Kg	mg/Kg	mg/Kg	mg/Kg	mg/Kg
	15	745	37	670	18	896	20
	30	463	25	342	5	480	3
	45	475	24	579	19	609	11
	60	391	20	502	17	379	10
	90	214	10	466	19	385	8
**February**	Malaxation time	Total Phenols mean	SD	Total Phenols mean	SD	Total Phenols mean	SD
	min	mg/Kg	mg/Kg	mg/Kg	mg/Kg	min	mg/Kg
	15	281	16	406	12	489	24
	30	289	11	315	14	511	13
	45	251	11	371	15	491	17
	60	290	9	333	3	438	18
	90	180	3	256	10	341	13

Table S6. Quantitation data for cv. Athenolia in 4 different harvest months, 5 different malaxation times at 28 ^{o}C

		**Oleocanthal**		**Oleacein**		**Oleokoronal**		**Oleomissional**		**Ligstroside Ag.**		**Oleuropein Ag.**		**Total Phenols**	
	Mal. Time (min)	mean	SD	mean	SD	mean	SD	mean	SD	mean	SD	mean	SD	mean	SD
**Nov**	15	99	4	80	10	687	33	371	18	41	3	42	0	1320	60
	30	150	9	92	4	492	11	284	18	43	3	45	2	1106	40
	45	195	13	99	9	427	34	264	10	53	3	43	2	1081	51
	60	224	17	95	9	427	13	147	3	57	2	50	3	1000	35
	90	270	10	82	8	297	11	60	0	76	3	50	2	835	26
**Dec**	15	67	5	61	2	632	38	352	13	20	0	30	1	1162	55
	30	152	6	83	10	315	10	69	4	22	1	35	0	676	27
	45	119	5	80	6	353	10	64	4	18	1	16	1	650	13
	60	106	13	75	9	436	16	137	11	22	2	16	1	792	42
	90	142	11	51	3	213	8	0	0	32	1	14	0	452	15
**Jan**	15	63	5	37	2	362	4	98	3	20	0	25	1	605	6
	30	78	6	39	0	334	12	88	9	27	1	30	0	596	20
	45	98	5	29	0	278	8	36	10	22	1	16	1	479	19
	60	99	7	30	2	120	4	25	11	27	2	18	1	319	12
	90	104	11	24	2	129	11	16	0	27	1	18	1	318	24
**Feb**	15	52	2	36	0	55	3	n.d.	0	n.d.	0	11	0	154	4
	30	55	3	32	2	27	0	n.d.	0	n.d.	0	n.d.	0	114	5
	45	57	2	30	2	27	3	n.d.	0	n.d.	0	n.d.	0	114	6
	60	60	4	20	2	29	3	n.d.	0	n.d.	0	n.d.	0	108	9
	90	60	3	7	0	22	0	n.d.	0	n.d.	0	n.d.	0	89	3

Table S7. Quantitation data for cv Throubolia in three different malaxation times at 28 °C

Malaxa-tion Time	Oleocanthal		Oleacein		Oleokoronal		Oleomissional		Ligstroside Ag.		Oleuropein Ag.		Total Phenols	
(min)	mean	SD	mean	SD	mean	SD	mean	SD	mean	SD	mean	SD	mean	SD
15	20	0	22	1	833	18	599	21	35	3	41	3	1550	36
30	34	2	28	2	600	26	521	28	95	6	46	3	1324	63
60	40	3	32	2	507	26	248	5	79	3	65	2	972	36

Table S8. Quantitation data for cv. Kalamata variety in three different malaxation times at 28 °C

Malax-ation Time	Oleocanthal		Oleacein		Oleokoronal		Oleomissional		Ligstroside Ag.		Oleuropein Ag.		Total Phenols	
(min)	mean	SD	mean	SD	mean	SD	mean	SD	mean	SD	mean	SD	mean	SD
**15**	748	52	202	9	20	1	20	1	5	0	5	0	1000	67
**30**	972	48	153	6	0	0	0	0	5	0	0	0	1130	66
**60**	947	27	98	3	0	0	0	0	0	0	0	0	1045	35

Table S9. Quantitation data for cv. Olympia in three different malaxation times at 28 °C

Malaxa-tion Time	Oleocanthal		Oleacein		Oleokoronal		Oleomissional		Ligstroside Ag.		Oleuropein Ag.		Total Phe-nols	
(min)	mean	SD	mean	SD	mean	SD	mean	SD	mean	SD	mean	SD	mean	SD
**15**	40	3	15	1	422	31	387	21	7	1	9	0	880	40
**30**	88	4	45	2	390	9	250	10	8	0	10	0	791	24
**60**	70	12	40	23	285	17	198	5	11	0	10	0	614	11

Table S10. Quantitation data for cv. Koroneiki variety in three different malaxation times at 28 °C

Malaxa-tion Time	Oleocanthal		Oleacein		Oleokoronal		Oleomis-sional		Ligstroside Ag.		Oleuropein Ag.		Total Phenols	
(min)	mean	SD	mean	SD	mean	SD	mean	SD	mean	SD	mean	SD	mean	SD
**15**	101	3	82	1	299	31	140	21	9	1	9	0	640	40
**30**	230	4	170	2	128	13	68	12	10	0	10	0	616	29
**60**	242	12	150	5	14	9	0	9	11	0	10	0	427	17

Table S11. The regression b coefficients and their statistically significance levels as R^2 of the linear regression model Total phenols = a + b*(malaxation time).

Month	Malaxation Temperature		
	**22°C**	**28°C**	**32°C**
**October**	b= -8.667 ***	b= -9.587 ***	b= -7.355 ***
	R^2= 0.926	R^2= 0.895	R^2= 0.895
**November**	b= -5.136 ***	b= -5.417 ***	b= -4.841 ***
	R^2= 0.857	R^2= 0.852	R^2= 0.814
**December**	b= -7.052 ***	b= -7.975 ***	b= -4.694 ***
	R^2= 0.723	R^2= 0.766	R^2= o.510
**January**	b= -6.205 ***	b= -1.323 ^{ns}	b= -5.801 ***
	R^2= 0.862	R^2= 0.095	R^2= 0.606
**February**	b= -1.258 ***	b= -1.632 ***	b= -2.171 ***
	R^2= 0.582	R^2= 0.661	R^2= 0.789

Table S12. The results of the analysis of variance (ANOVA) and Duncan's MRT at *p*= 0.05 for oleocanthal.

Month	Malaxation Temperature	Malaxation Time		
		15 min	30 min	45 min
October	32	208[a]	254[a]	237[b]
	28	213[a]	254[a]	264[a]
	22	90[b]	117[b]	139[c]
	p	***	***	***
November	32	136[a]	254[a]	267[a]
	28	150[a]	188[b]	195[b]
	22	53[b]	99[c]	106[c]
	p	***	***	***
December	32	144[a]	168[a]	201[a]
	28	96[b]	150[a]	190[a]
	22	44[c]	88[b]	114[b]
	p	***	***	***
January	32	116[a]	106[a]	160[a]
	28	68[b]	67[b]	124[b]
	22	39[c]	67[b]	83[c]
	p	***	**	***
February	32	78[a]	72[a]	85[a]
	28	52[b]	63[b]	53[b]
	22	21[c]	42[c]	45[c]
	p	***	***	***

The means followed by the same letter in each month (a, b and c as superscript) are not statistically different.

Table S13. The results of the analysis of variance (ANOVA) and Duncan's MRT at p= 0.05 for Oleacein.

**Month**	**Malaxation Temperature**	**Malaxation Temperature**		
		**15 min**	**30 min**	**45 min**
**October**	32	95^{b}	56^{b}	24^{c}
	28	112^{a}	68^{a}	42^{a}
	22	59^{c}	56^{b}	36^{b}
	p	**	*	**
**November**	32	130^{a}	201^{a}	188^{a}
	28	135^{a}	138^{b}	119^{b}
	22	48^{b}	51^{c}	53^{c}
	p	***	***	***
**December**	32	147^{α}	198^{a}	195^{a}
	28	123^{b}	147^{b}	169^{b}
	22	56^{c}	80^{c}	92^{c}
	p	***	***	***
**January**	32	114^{a}	109^{a}	107^{a}
	28	58^{b}	53^{b}	89^{b}
	22	49^{b}	42^{c}	48^{c}
	p	***	***	***
**February**	32	112^{a}	126^{a}	133^{a}
	28	70^{b}	73^{b}	87^{b}
	22	29^{c}	51^{c}	49^{c}
	p	***	***	***

The means followed by the same letter in each month (a, b and c as superscript) are not statistically different.

TableS14. Sampling and experimental planning

Cultivar	Region	Harvest Month	Malaxation Temperature (^oC)	Malaxation Time (min)
**Koroneiki**	Molaoi	October	22,28,32	15,30,45,60,90
		November	22,28,33	15,30,45,60,90
		December	22,28,34	15,30,45,60,90
		January	22,28,35	15,30,45,60,90
		February	22,28,36	15,30,45,60,90
**Athenolia**	Molaoi	November	28	15,30,45,60,90
		December	28	15,30,45,60,90
		January	28	15,30,45,60,90
		February	28	15,30,45,60,90
**Throubolia**	Naxos	October	28	15,30,60
**Olympia**	Ilia	October	28	15,30,60
**Koroneiki**	Kalamata	October	28	15,30,60
**Kalamata**	Sparta	October	28	15,30,60
